# Diurnal changes of arterial oxygen saturation and erythropoietin concentration in male and female highlanders

**DOI:** 10.14814/phy2.12901

**Published:** 2016-09-05

**Authors:** Edgar Cristancho, Alain Riveros, Armando Sánchez, Oscar Peñuela, Dieter Böning

**Affiliations:** ^1^ Departamento de Biologia Division de Fisiologia Animal Universidad Nacional de Colombia Bogotá Colombia; ^2^ Pontificia Universidad Javeriana Bogotá Colombia; ^3^ Charité – Universitätsmedizin Berlin Germany

**Keywords:** Circadian rhythm, female hormones, hypoxia, respiration, sex difference

## Abstract

In Caucasians and Native Americans living at altitude, hemoglobin mass is increased in spite of erythropoietin concentrations ([Epo]) not markedly differing from sea level values. We hypothesized that a nocturnal decrease of arterial oxygen saturation (SaO_2_) causes a temporary rise of [Epo] not detected by morning measurements. SaO_2_ (continuous, finger oximeter) and [Epo] (ELISA, every 4 h) were determined in young highlanders (altitude 2600 m) during 24 h of usual daily activity. In Series I (six male, nine female students), SaO_2_ fell during the night with the nadir occurring between 01:00 and 03:00; daily means (range 92.4–95.2%) were higher in females (+1.7%, *P* < 0.01). [Epo] showed opposite changes with zenith occurring at 04:00 without a sex difference. Mean daily values (22.9 ± 10.7SD U/L) were higher than values obtained at 08:00 (17.2 ± 9.5 U/L, *P* < 0.05). In Series II (seven females), only SaO_2_ was measured. During follicular and luteal phases, SaO_2_ variation was similar to Series I, but the rhythm was disturbed during menstruation. While daily [Epo] variations at sea level are not homogeneous, there is a diurnal variation at altitude following changes in SaO_2_. Larger hypoventilation‐dependent decreases of alveolar PO_2_ decreases during the night probably cause a stronger reduction of SaO_2_ in highlanders compared to lowlanders. This variation might be enlarged by a diurnal fluctuation of Hb concentration. In spite of a lower [Hb], the higher SaO_2_ in women compared to men led to a similar arterial oxygen content, likely explaining the absence of differences in [Epo] between sexes.

## Introduction

Erythropoietin concentration ([Epo]) rises during ascent to altitude, but soon decreases and returns to preceding or only slightly increased values after more than 30 days (Siebenmann et al. 2015; Böning et al. 1997; Lundby et al. 2014). The topic has been reviewed by Klausen (1998), Gunga et al. (2007), and Jelkmann (2011). Possible mechanisms might be down‐regulation of the hypoxia‐inducible factor alpha (HIF*α*) or degradation of Epo by the target cells in the bone marrow (Jelkmann 2011). In native highlanders, there are only few investigations on Epo. Winslow et al. (1989) and Schmidt et al. (1993) did not find an increase compared to lowlanders above 3500 m of altitude in the Himalaya as well as in the Andes. The [Epo] increase was also small or absent at 2600 m in Bogotá (Böning et al. 2001, 2004; Schmidt et al. 2002; Cristancho et al. 2007). In contrast to Tibetans, genetic adaptation of HIF*α* probably does not play a role in Epo secretion among Andean natives (Bigham et al. 2009). There are, however, changes in HIF‐targeted genes important for metabolism in an oxygen‐deprived cellular environment and for microcirculation.

In spite of the lack of rise in [Epo], Hb concentration as well as Hb mass (or the corresponding red cell volume) are increased in altitude visitors and in South American dwellers (reviewed in Sawka et al. 2000; West et al. 2007). Various investigations have shown that Hb mass in most visitors continues to rise in spite of the normalization of [Epo] after about 30 days at altitude (e.g., Milledge and Cotes 1985; Pugh 1964). Shaw and Simpson (1961) reviewed articles up to 1961 and found a rise of approximately 50% in red cell mass at 5000 m of altitude. Weil et al. (1968) measured a clear increase by nearly 5 mL/kg body mass (approx. 18%) at 3100 m in male North American highlanders. Various studies were performed on inhabitants of Bogotá using the CO rebreathing method to measure Hb mass. Hb mass was found to be increased by 12 to 22% in males (Böning et al. 2001; Schmidt et al. 2002) and by less than 7% in females (Böning et al. 2004) when compared to lowlanders.

The stimulus for Epo secretion is a decrease in arterial oxygen content leading to lowered tissue PO_2_ in the kidney (Jelkmann 2011). Weil et al. (1968) observed an inflection point on the arterial PO_2_ red cell mass curve in highlanders at approx. 67 mmHg P_a_O_2_ corresponding to 93–94% arterial oxygen saturation (SaO_2_). This indicates the “shoulder” of the oxygen dissociation curve (ODC) where a further small reduction of PO_2_ causes a marked fall of saturation (Sutton et al. 1980). In some subjects, the arterial P_a_O_2_ falls below the threshold already at 1600 m of altitude (Weil et al. 1968); at 2600 m, about half of the investigated young male and female inhabitants show values below the threshold (Böning et al. 2001, 2004).

In most investigations, [Epo] as well as SaO_2_ were measured once a day. But this can only be representative if there are no diurnal changes. Until now it is not clear whether a circadian rhythm for the secretion of the hormone exists (Cahan et al. 1992; Wide et al. 1989; Pasqualetti et al. 1996, 2000; Gunga et al. 2003, 2007); perhaps, physical activity and illness play a modifying role (Klausen 1998). While some authors (Gunga et al. 2003, 2007) observed no circadian rhythm during bed rest at sea level, others (Fitzpatrick et al. 1993; Klausen et al. 1996) detected a mostly small nocturnal [Epo] increase in subjects with usual daily activity.

Interestingly, during a short altitude stay (64 h at 4350 m), Klausen et al. (1996) measured an enlarged variation in [Epo] with peak values at 04:00 (+6 U/l compared to 08:00). The stimulation of ventilation by increased PCO_2_ and decreased PO_2_ in arterial blood is reduced during the night (reviewed by Buchanan 2013). It is conceivable that SaO_2_ decreases during night sleep and leads to a rise in mean 24‐h concentration of Epo causing the enlarged Hb mass. Unfortunately, Klausen et al. (1996) did not communicate SaO_2_ at various times of the day. But Fitzpatrick et al. (1993) as well as Gries and Brooks (1996) observed that already at sea level, saturation decreases during sleep in healthy subjects. This effect should be exaggerated at altitude when arterial PO_2_ reaches the “shoulder” of the ODC. Indeed, SaO_2_ decreases during sleep in healthy people living at 4380 m from approximately 86% in the awake state to a nadir of 83% between 01:00 and 03:00 (Spicuzza et al. 2004).

An additional interesting question is whether there are sex‐specific differences in SaO_2_ and correspondingly in [Epo]. Ventilatory stimulation by female sex hormones might cause a reduced decrease of SaO_2_ in women at altitude (Böning et al. 2001, 2004; Cristancho et al. 2007). Beall et al. (1997); Beall (2000b) measured daytime SaO_2_ in highlanders in Tibet and Bolivia. In Tibet, at an altitude of approx. 4000 m, SaO_2_ was significantly higher in fertile females than in males of equal age (Beall 2000a). For clarification, it seems useful to compare whole‐day profiles of SaO_2_ in both sexes.

This study addresses the following questions:

1. Is there a diurnal rhythm of [Epo] in the blood of highlanders with increased nocturnal values which might be the cause for increased Hb concentration as well as Hb mass?
2. Is this hypothetical rhythm caused by corresponding variations in arterial SaO_2_?
3. Are there sex differences in arterial SaO_2_ and correspondingly in [Epo] in highlanders?

## Methods

The investigations were performed in Bogotá (2600 m above sea level) in subjects born at this altitude or living there for at least 3 years. Most inhabitants of this region are mestizos (Sandoval et al. 1993). Fifteen nonsmoking untrained medical students (six men, 19.4 ± 2.1 years, 66.7 ± 8.7 kg, 176.2 ± 6.8 cm, and nine women, 18.9 ± 0.7 years, 52.5 ± 6.2 kg, 156.7 ± 8.6 cm; means ± standard deviations) participated in Series I, in which [Epo] and SaO_2_ determinations were carried out. In Series II, only SaO_2_ was measured in another group of seven untrained nonsmoking female students (age 22.1 ± 1.64 years, body mass 52.2 ± 3.8 kg, height 163 ± 5 cm) during different phases of the menstrual cycle. Informed consent was obtained from all participants and experimental protocols were approved by the ethics committee of the Science Faculty of the Universidad Nacional de Colombia. All subjects were clinically healthy. They performed only lessons and laboratory work during the experimental time, heavy physical activity and sports were not allowed.

### Study design

#### Series I

The subjects reported to the laboratory at 07:45 for preparation and anthropological measurements. SaO_2_ was measured continuously during 24 h with a finger oximeter (Wrist‐Ox 3100, Nonin, Minneapolis, MN). As the oximeter could not be checked continuously throughout the day, values were occasionally lost; in two females, sufficient data were not obtained. Blood samples were withdrawn from subjects in a seated position into EDTA vacutainer tubes every 4 h from 08:00 to 04:00 of the next day. In the first sample, [Hb] was measured with the cyanmethemoglobin method and hematocrit (Hct) values were calculated from [Hb] and mean red cell volume (ADVIA 120 hematology analyzer, Bayer Diagnostics, Tarrytown, NY). [Epo] was determined in plasma with a commercial ELISA kit (R&D systems, Minneapolis, MN). The samples were stored at −20°C maximally for 1 month. During the day, the subjects performed their usual tasks as students in the Universidad Militar, and at night, they slept from 22:00 to 07:00 in the university hospital to allow for the withdrawal of the blood samples. They took their meals after blood sampling.

#### Series II

SaO_2_ measurements were performed for 24 h during menstruation, luteal phase (14 days before the onset of menstruation) and follicular phase (remaining days). All subjects stated they were not taking contraceptives.

### Calculations

#### Statistics

The data are presented as means ± standard deviations (SD) in the text, ± standard errors (SE) in the figures. Depending on the number of comparisons, *t*‐tests or analysis of variance (ANOVA) with subsequent Bonferroni corrected *t‐*tests were used for significance calculations (two‐tailed). Linear regression and correlation coefficients served as indicators for a relation between SaO_2_ and [Epo] considering the delay in secretion of the latter. Calculations were performed with SPSS (version 19).

## Results

### Diurnal changes of arterial oxygen saturation and erythropoietin concentration in male and female highlanders (Series I)

**Figure 1:**
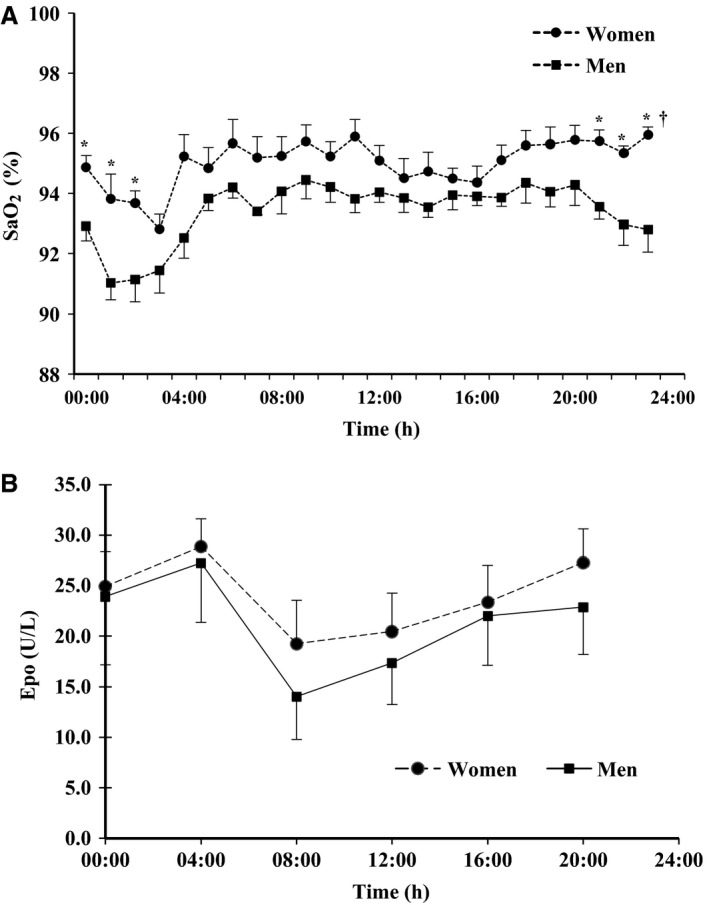
Diurnal changes of arterial oxygen saturation (SaO_2_, A) and erythropoietin (Epo) concentration (B) in six men (squares) and nine women (filled circles). Means and SE. SaO_2_ was only measured in five males and seven females. Asterisks indicate significant differences between sexes for single hours (*P *< 0.05 or better).

The male and female subjects presented normal sex differences and a slight altitude effect in hematological quantities (Males: [Hb] 16.4 ± 0.7 g/dL, Hct 46.6 ± 3.2%; females: [Hb] 14.8 ± 0.9 g/dl, Hct 45.1 ± 2.7%). Figure 1 shows SaO_2_ during 24 h (upper panel). There are two remarkable aspects. Firstly, SaO_2_ varied in both sexes with nadir occurring between 01:00 and 03:00 (ANOVA for all subjects: *P *<* *0.001). Secondly, individual daily means differed markedly (range from 92.4 to 95.2%). At any time of the day, women had a higher oxygen saturation than men (overall *P *<* *0.01). On average, the difference amounts to 1.7% but increases up to 3.2% during the night (*P* at least <0.05 from 21:00 to 03:00 for each hour). SaO_2_ decreased in men earlier in the evening (from 20:00 onwards); however, the maximal change was similar in both sexes (approx. 3.0%). From 04:00 to 06:00, SaO_2_ recovered with a similar pattern in both sexes and then varied little until 20:00.

[Epo] (Fig. 1, lower panel) also showed diurnal changes (ANOVA *P *<* *0.02 for all subjects), but no significant difference between sexes. The variations were inverse and slightly delayed with regard to those of arterial saturation in the period between 00:00 and 08:00. When SaO_2_ was decreased (00:00 to 03:00), [Epo] reached highest values at 04:00, and when SaO_2_ increased (04:00–06:00), [Epo] decreased to the lowest value at 08:00. In the following hours, while SaO_2_ variations were small, [Epo] slightly increased (not significant). A comparison of the mean value for all subjects at 08:00 (17.2 ± 9.5 U/L) with the rest of the day (mean: 24.0 ± 11.4 U/L) or the whole day (24 h mean: 22.9 ± 10.7 U/L) yielded a significant difference (all *P* < 0.05). Thus, the mean diurnal value is one‐third higher than the morning value.

### Correlations

**Figure 2:**
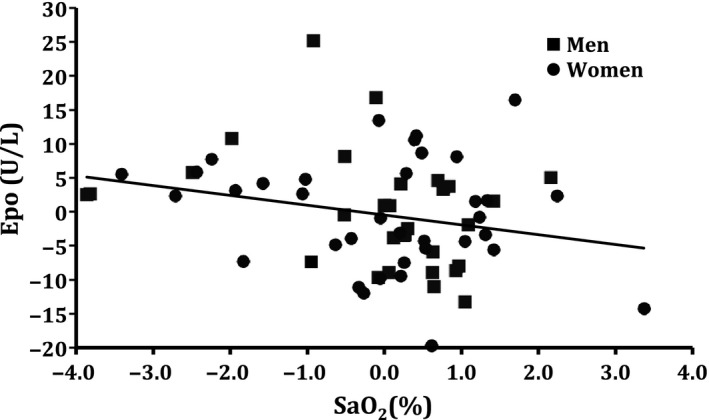
Correlations between changes in arterial oxygen saturation (SaO_2_) and [Epo]. SaO_2_ averaged 1–3 h before blood sampling in males, and 1 h in females. Common regression equation: [Epo] = −1.5 (SO_2_) −0.5; *r *= −0.25 (*P *< 0.05).

Correlations between SaO_2_ and [Epo] were calculated for the subgroups. Significant negative coefficients were obtained only for males with highest values for SaO_2_ 1–3 h before blood sampling for Epo determination (*r* = −0.47, *P *<* *0.01). For females, no significant values were found (best *r* = −0.02 for SaO_2_ 1–2 h before blood sampling). However, when deviations from the daily means were calculated, the common regression for the sex‐specific nadir SaO_2_ was significant (Fig. 2).

### Diurnal changes of arterial oxygen saturation in female highlanders in relation to the menstrual cycle (Series II)

**Figure 3:**
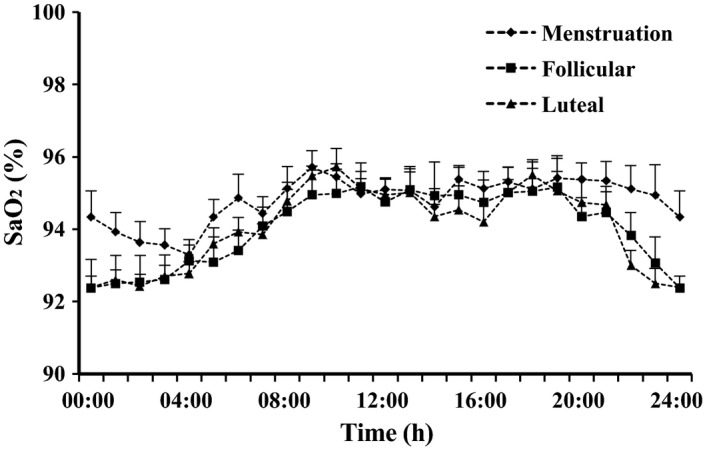
Diurnal changes of arterial oxygen saturation (SaO_2_) in seven women during the follicular phase (squares), the luteal phase (triangles), and menstruation (diamonds). Means and SE.

The course of SaO_2_ was equal during the follicular and luteal phases and similar to that in Series I, but with a more marked and longer decrease during the night between 22:00 and 08:00 with lowest values around midnight (ANOVA *P* < 0.001). During the menses, however, the nocturnal depression was only moderate with the nadir at 04:00 (*P* < 0.01). The time course of the SaO_2_ variation was significantly different from those during the other phases (*P* < 0.01) (Fig. 3).

## Discussion

### General

As assumed, we found a diurnal rhythm of [Epo] in highlanders at moderate altitude following arterial oxygen saturation changes with peak values occurring after the nocturnal decrease of SaO_2_. Changes in SaO_2_ and [Epo] correlated significantly. In spite of a generally higher SaO_2_, there was no lower [Epo] in females.

### Diurnal changes of arterial oxygen saturation and erythropoietin concentration in male and female highlanders (Series I)

The highlanders at 2600 m above sea level showed a clear nocturnal decrease of SaO_2_ with the nadir occurring between 01:00 and 03:00, similar to changes seen at 4380 m (Spicuzza et al. 2004). The values throughout 24 h varied between approximately 96 and 91%. According to Weil et al. (1968), the threshold for the stimulation of Epo secretion is located within this range of arterial saturations. Because of the “shoulder” of the ODC, equal diurnal differences in arterial PO_2_ cause larger SaO_2_ variations at altitude than at sea level. When the arterial PO_2_ decreases more markedly in chronic mountain sickness, [Epo] fluctuations are exaggerated, but a diurnal rhythm ceases to exist (Bernardi et al. 2003).

The suggested correlations between SaO_2_ and [Epo] were better in males than in females. The lower *r* values in females might be caused by larger scattering (perhaps caused by dysmenorrhea in some subjects, see below) and by the fact that saturation measurements were less complete leading to a loss of 13 pairs of data.

Decisive for Epo secretion, however, is the tissue PO_2_ in the kidneys which in turn depends on arterial PO_2_, [Hb], and oxygen affinity, while blood flow plays a minor role (Jelkmann 2011, 2013). These three quantities determine the oxygen content of arterial blood, which also can be calculated as SaO_2_ (as fraction) times [Hb] times 1.34 mL O_2_ **/**g Hb; the latter is the maximal binding capacity considering a small amount of CO‐Hb and MetHb. While arterial PO_2_ and affinity decrease at moderate altitude, [Hb] rises thus stabilizing the arterial oxygen content. When comparing residents in Berlin (30 m above sea level) and Bogotá (Böning et al. 2001, 2004), there is even a tendency for an increased arterial oxygen content in Bogotá at approximately 08:00: 20.1 and 21.7 mL/dL for males, 16.7 and 18.0 mL/dL for females. Additionally, the oxygen dissociation curve is slightly right‐shifted in Bogotá (approx. +2 mmHg for standard P_50_) causing a small increase in PO_2_ at any given SaO_2_ (Schmidt et al. 1990). This explains why no substantial increase in [Epo] was found for highlanders as compared with lowlanders in blood sampled during the morning in various studies in Bogotá but also at higher sites (Winslow et al. 1989; Böning et al. 2001, 2004; Schmidt et al. 2002; Cristancho et al. 2007).

**Figure 4:**
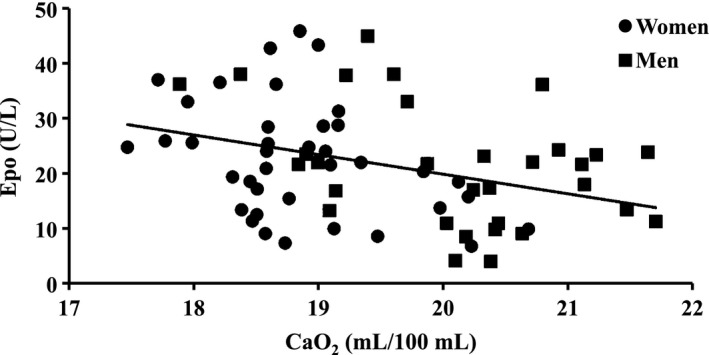
Correlation between calculated arterial oxygen content (CaO_2_) 1–3 h before venous blood sampling and [Epo]. Oxygen content is calculated assuming a diurnal [Hb] variation as at sea level (Böning et al. 1974). Regression equation: [Epo] = −3.6 C_a_O_2_ + 91 (*r *= 0.35, *P *< 0.02).

At sea level also [Hb] shows a diurnal variation (Böning et al. 1974; Wisser and Breuer 1981) with lower values occurring during the night (amplitude approximately 4%), probably because of reduced filtration in the leg vessels in supine position. If such a rhythm exists at altitude as well, the [Epo] increase during the night should be larger than expected only from the mean saturation decrease of 3%. We have estimated the change in arterial O_2_ content assuming a variation in [Hb] as at sea level (Fig. 4). Calculation of the correlation between O_2_ content and [Epo] yielded significant dependencies for both males and females. For all subjects, the regression equation indicates an [Epo] increase of 3.6 U/L per 1 mL/dL decrease in O_2_ content during 1–3 h before blood sampling. As an acute hypoxic stimulus on Epo secretion needs 80–120 min to be effective (Eckardt et al. 1989), the diurnal variation probably is caused by the preceding changes of both SaO_2_ and [Hb].

At first glance surprisingly, [Epo] is not decreased in females compared to males in spite of the increased SaO_2_ in the former group. But again the Hb concentration plays a role. Because of the lower [Hb] in women (−9%) the arterial O_2_ content is reduced, and thus the stimulus for Epo secretion is even slightly increased (women: arterial oxygen content at 08:00 18.9 vs. 20.4 mL/dL in males). As Hb mass is finally determined by production and destruction of erythrocytes, this quantity is lowered in young women compared to men partly by the regular loss of blood during menstruation in spite of a high [Epo]. Another mechanism for the general Hb mass difference between sexes is the erythropoietic effect of androgens (Murphy et al. 2010).

### Diurnal changes of arterial oxygen saturation in female highlanders in relation to the menstrual cycle (Series II)

The nocturnal decrease of SaO_2_ lasted longer than in Series I except during menstruation. Possibly, the undisturbed sleep without blood sampling was the cause; the subjects had refused the use of indwelling cannulas in the series with [Epo] measurements. On the other hand, dysmenorrheal troubles might have reduced sleep quality during menstruation (Iacovides et al. 2015) and hindered the reduction of ventilation between 22:00 and 03:00. From these observations, one might suggest that the nocturnal increases of [Epo] are in reality larger than measured in Series I.

The curves for SaO_2_ during the follicular and luteal phases are indistinguishable corresponding to a study of Reeves et al. (2001) who did not find cycle‐dependent differences in SaO_2_ or [Epo]. This is not fully unexpected because a larger stimulating effect of progesterone rather than estrogen on respiration has been described occasionally, but was not uniformly observed (reviewed in Cristancho et al. 2007 and MacNutt et al. 1985). The mechanism of the altered ventilatory drive in females is still not known; the hormones possibly act on the carotid body and respiratory centers in the brain stem_._ MacNutt et al. (1985) have described the CO_2_ threshold for the stimulation of ventilation as being always lower in females than in males. A rise in energy metabolism during the luteal phase might also contribute to the increase in ventilation. As contraceptive drugs contain estrogen‐ and progesterone‐like substances, the sex difference should be observable in all women before menopause.

## Conclusions

This study shows the presence of a diurnal rhythm in serum Epo levels with highest values at approx. 04:00 in highlanders with normal daily activity. Morning [Epo] is about one‐third lower than the mean value for 24 h. The mechanism causing the diurnal rhythmicity is a variation in arterial oxygen content. For assessment of serum Epo values, the time of day for collection of blood samples has to be taken into consideration.

Larger hypoventilation‐dependent arterial SO_2_ decreases during the night probably cause a stronger reduction of arterial O_2_ content in highlanders compared to lowlanders causing a higher Epo secretion and thus Hb mass in the former.

SaO_2_ is higher in females than males especially during sleep at night. The rhythm does scarcely vary during different phases of ovarian function, except during menstruation. In spite of the difference in SaO_2_, a difference between sexes in Epo concentration could not be detected because of the lowered [Hb] in women; this resulted in similar arterial O_2_ content in males and females.

## Conflicts of Interest

None declared.
